# Clinical Outcomes and Risk Factors of Healthcare-Associated Infections in Surgical Wards: A Retrospective Cohort Study

**DOI:** 10.3390/medicina62050995

**Published:** 2026-05-20

**Authors:** Andreea Mihaela Sandu, Corneliu Ovidiu Vrancianu, Marian Necula, Roxana-Elena Cristian, Ana-Catalina Tantu, Alina Păunescu, Daniel Diaconescu, Monica Marilena Tantu

**Affiliations:** 1 Doctoral School, Carol Davila University of Medicine and Pharmacy, Eroii Sanitari 8, District 5, 050474 Bucharest, Romania; andreea-mihaela.sandu@drd.umfcd.ro; 2 The County Emergency Hospital, Aleea Spitalului 36, 110283 Pitești, Romania; 3 National Institute of Research and Development for Biological Sciences, 296 Splaiul Independentei, District 6, 060031 Bucharest, Romania; marian.necula@incdsb.ro (M.N.); roxana.cristian@incdsb.ro (R.-E.C.); 4 Department of Microbiology and Immunology, The Research Institute of the University of Bucharest, ICUB, Șoseaua Panduri 90, District 5, 050663 Bucharest, Romania; 5 Faculty of Administration and Business, University of Bucharest, Regina Elisabeta 4-12, 030167 Bucharest, Romania; 6 Doctoral School, University of Medicine and Pharmacy of Craiova, Petru Rareș 2, 200349 Craiova, Romania; ana.tantu@umfcv.ro; 7 Emergency Clinical County Hospital of Bucharest, Calea Floreasca 8, 014461 Bucharest, Romania; 8 Department of Natural Sciences, Faculty of Sciences, Physical Education and Informatics, National University of Science and Technology POLITEHNICA Bucharest, Pitești University Center, Targu din Vale Street 1, 110040 Pitesti, Romania; alina.paunescu@upb.ro; 9 Department of Medical Assistance and Physical Therapy, Faculty of Sciences, Physical Education and Informatics, National University of Science and Technology POLITEHNICA Bucharest, Pitești University Center, Targu din Vale Street 1, 110040 Pitesti, Romania; daniel.diaconescu@upb.ro (D.D.); marilena.tantu@upb.ro (M.M.T.)

**Keywords:** healthcare-associated infections, *Clostridioides difficile* infection, SARS-CoV-2 infection, in-hospital mortality, comorbidity burden

## Abstract

*Background and Objectives*: Healthcare-associated infections (HAIs) remain a major cause of morbidity and mortality among hospitalized patients. During the COVID-19 pandemic, SARS-CoV-2 infection emerged as a major contributor to HAIs, alongside *Clostridioides difficile* infection (CDI) and other bacterial infections. This study aimed to evaluate the clinical characteristics and outcomes of HAIs in surgical departments and to identify factors associated with in-hospital mortality. *Materials and Methods*: We conducted a retrospective observational study including 170 patients with documented HAIs admitted between July 2018 and June 2022 in surgical departments of a county emergency hospital. Patients were categorized into SARS-CoV-2 infection (*n* = 85), CDI (*n* = 73), and other bacterial infections (*n* = 12), the latter being included for descriptive purposes only due to limited sample size. Clinical variables, comorbidities, prior antibiotic exposure, length of hospital stay, and in-hospital mortality were analyzed. Survival analysis and logistic regression were performed to identify predictors of mortality. *Results*: SARS-CoV-2 infection represented the largest subgroup, followed by CDI. Overall, in-hospital mortality was 15.9%, with comparable rates between SARS-CoV-2 infection (17.6%) and CDI (16.4%), while no deaths were observed in the small subgroup of other bacterial infections. CDI patients had a significantly higher burden of comorbidities (*p* = 0.004). Kaplan–Meier analysis did not show a statistically significant difference in survival between SARS-CoV-2 and CDI groups (log-rank *p* = 0.28). In univariate analysis, acute respiratory failure (OR ≈ 13.5, *p* < 0.001), chronic kidney disease (OR ≈ 4.4, *p* = 0.018), and number of comorbidities (*p* = 0.019) were associated with mortality, but none remained significant in multivariable analysis. *Conclusions*: In-hospital mortality was similar between SARS-CoV-2 infection and CDI, highlighting the persistent clinical impact of CDI in hospitalized patients. Comorbidity burden and acute complications, particularly respiratory failure, were key determinants of mortality. These findings highlight the persistent clinical impact of CDI and the role of comorbidity burden and acute complications, particularly respiratory failure, in shaping in-hospital mortality. The absence of independent predictors in multivariable analysis should be interpreted cautiously given the limited sample size.

## 1. Introduction

Healthcare-associated infections (HAIs) remain a major cause of morbidity and mortality across healthcare settings worldwide. A recent large prospective European cohort study reported that more than one in two residents in long-term care facilities experienced at least one HAI, with a considerable burden of hospitalization and mortality [1]. Beyond their clinical impact, HAIs are also associated with prolonged hospital stay and increased healthcare costs, particularly among vulnerable populations such as patients with cancer or severe infections [2]. Despite advances in infection prevention and control, HAIs continue to represent a significant burden, especially in surgical settings where invasive procedures and prolonged hospitalization increase the risk of infection.

Among HAIs, *Clostridioides difficile* infection (CDI) is one of the leading causes of nosocomial diarrhea and is associated with substantial morbidity and mortality. The incidence and severity of CDI have increased in recent years, particularly among elderly and comorbid patients [3]. Recent clinical evidence further highlights the role of CDI in severe and recurrent disease, with mortality closely linked to advanced age and underlying comorbidities [4]. In addition to patient-related risk factors, environmental contamination plays a key role in CDI transmission, with studies reporting significant prevalence of CDI in healthcare settings and on medical equipment [5,6].

HAIs are particularly prevalent among critically ill patients, with systematic evidence demonstrating that pneumonia, bloodstream infections, and urinary tract infections represent the most common types. For instance, studies conducted in neurosurgical settings have shown that pneumonia, bloodstream infections, and surgical site infections are among the most frequent HAIs, particularly in patients undergoing emergency procedures or presenting with severe clinical status [7]. A recent meta-analysis reported high pooled prevalence rates for ventilator-associated pneumonia and other major HAIs in adult intensive care units, highlighting the role of invasive procedures, comorbidities, and antimicrobial exposure as key risk factors [8].

The emergence of the coronavirus disease 2019 (COVID-19) pandemic has further complicated the epidemiology of HAIs. Severe acute respiratory syndrome coronavirus 2 (SARS-CoV-2) infection has not only increased hospitalization rates but has also influenced antimicrobial use, infection control practices, and the incidence of secondary infections. Patients hospitalized with COVID-19 are particularly susceptible to secondary infections, which are associated with prolonged hospitalization and increased mortality [9]. Growing evidence suggests that the pandemic has modified HAI patterns, including changes in incidence, pathogen distribution, and antimicrobial resistance (AMR) profiles, although findings remain heterogeneous across settings [10,11,12,13]. In this context, Gram-negative pathogens such as *Klebsiella* spp., *Acinetobacter* spp., and *Pseudomonas* spp. have been frequently reported as dominant causative agents, often associated with increasing AMR during the pandemic period [11,13].

Moreover, HAIs have been shown to significantly worsen outcomes in patients with COVID-19, contributing to increased mortality and more complex clinical courses. Large-scale analyses indicate that HAIs occurrence is higher among hospitalized COVID-19 patients compared to non-COVID populations, with substantially increased rates of device-associated infections such as bloodstream and urinary tract infections [2,10]. National registry data further confirm that HAI-related mortality is consistently higher among infected patients, particularly in the early stages of hospitalization [14]. A Romanian retrospective study reported a mortality rate of 32.5% among patients with COVID-19 and HAIs, with advanced age and surgical factors identified as independent predictors of death [15].

In parallel, the growing burden of AMR continues to impact the clinical course of HAIs. Studies conducted in Romania and other regions have highlighted high rates of multidrug-resistant organisms and their association with adverse clinical outcomes [16,17]. At the same time, infection prevention and control measures implemented during the COVID-19 pandemic have shown variable effectiveness, with some studies reporting reductions in certain types of HAIs, while others observed stable or even increased rates depending on the clinical context [18].

Despite the increasing number of studies addressing HAIs, limited data are available on the comparative clinical outcomes of different infection categories, particularly in surgical departments where patient vulnerability is high. Furthermore, the interplay between infection type, comorbidity burden, and mortality risk remains insufficiently explored. Therefore, this study aimed to evaluate the epidemiological, clinical, and microbiological characteristics of HAIs in surgical departments and to assess their association with hospitalization duration and in-hospital mortality.

## 2. Materials and Methods

### 2.1. Study Design and Ethics

This was a retrospective, single-center cohort study conducted in the surgical departments of The County Emergency Hospital in Pitesti, Romania between July 2018 and June 2022. The study period included the COVID-19 pandemic, which substantially affected hospital organization, surgical activity, and infection control practices, including reduced elective procedures, modified patient flow, and changes in case-mix leading to more urgent and severe conditions.

This study was conducted in accordance with ethical standards and was approved by the Ethics Subcommittee for Scientific Research of the Carol Davila University of Medicine and Pharmacy [19].

### 2.2. Study Setting

The analysis focused on infections occurring in surgical departments of a county emergency hospital over a four-year period. The study included multiple surgical wards, namely general surgery I and II, orthopedics, urology, neurosurgery, vascular surgery, plastic surgery, otorhinolaryngology, and obstetrics and gynecology.

During the COVID-19 pandemic, surgical activity in these departments was partially restructured, with a reduction in elective procedures and prioritization of urgent cases, which may have influenced the observed distribution of infections.

### 2.3. Study Population

The study population consisted of 170 hospitalized patients with infections identified during hospitalization in surgical departments. Cases were categorized into three infection groups: SARS-CoV-2 infection (*n* = 85), CDI (*n* = 73), and other microbiologically confirmed bacterial infections (*n* = 12).

Given the limited size of the “other bacterial infections” subgroup, this category was considered for descriptive purposes only and was excluded from comparative statistical analyses to avoid underpowered and potentially misleading results. The primary comparative analyses were therefore focused on SARS-CoV-2 infection and CDI.

Patients were eligible for inclusion if they were admitted to a participating surgical ward and developed a clinically and laboratory-confirmed infection during hospitalization, in accordance with institutional surveillance protocols. Case identification was based on routinely collected clinical and epidemiological data.

Bacterial infections were included only if microbiological confirmation and antimicrobial susceptibility testing (AST) results were available. Only isolates with complete susceptibility profiles were considered for microbiological characterization. Multidrug resistance (MDR) was defined as non-susceptibility to at least one agent in three or more antimicrobial classes.

Patients with evidence of colonization or asymptomatic carriage, as well as cases without a clear epidemiological link to the hospitalization episode, were excluded. Cases with incomplete medical records or insufficient linkage between clinical and microbiological data were also excluded.

The final study cohort of 170 patients was derived from a total of 37,482 hospital admissions across the participating surgical departments between July 2018 and June 2022. Cases were identified through clinical records and infection surveillance databases after applying predefined inclusion and exclusion criteria.

### 2.4. Data Sources and Data Collection

The dataset was constructed from hospital electronic medical records, microbiology laboratory databases, and institutional infection surveillance registries.

Extracted variables included demographic data (age, sex), clinical characteristics (comorbidities, admission diagnosis), hospitalization data (length of stay—LOS, time to infection detection), and outcomes (in-hospital mortality).

For CDI cases, laboratory confirmation was based on glutamate dehydrogenase (GDH) antigen detection and toxin A/B assays, in accordance with institutional diagnostic protocols. Information on antibiotic exposure at admission was available and included in the analysis.

SARS-CoV-2 infection was defined based on laboratory-confirmed diagnosis, as recorded in hospital clinical databases.

For other bacterial infections, microbiological data included culture-confirmed isolates with available antimicrobial susceptibility testing (AST) results. Antibiotic resistance profiles (sensitive/resistant) were available for this subgroup and were used for descriptive characterization only, given the limited sample size.

Due to heterogeneity in microbiological data availability across infection categories, pathogen-specific resistance profiles and antibiotic exposure were not uniformly analyzed across all groups but were incorporated where data were available to enhance microbiological context.

To address the temporal relationship between hospitalization and infection occurrence, the time to infection detection was calculated for all cases. This allowed partial differentiation between infections present at admission and those developing during hospitalization.

### 2.5. Variables and Definitions

Demographic variables included age, sex, and living environment. Clinical variables comprised the admitting department and comorbidities, which were recorded both individually and as a comorbidity burden expressed as the total number of comorbid conditions per patient, as well as a binary variable indicating the presence of any comorbidity.

Hospitalization-related variables included LOS and time from admission to infection detection. Exposure variables included hospitalization within the previous 60 days, antibiotic exposure within 60 days, and antibiotic treatment at admission.

Infection-related variables included infection category, microbiological findings, CDI diagnostic markers (glutamate dehydrogenase antigen and toxin A/B detection), and pathogen distribution in bacterial infections.

HAIs were defined according to CDC/ECDC criteria as infections occurring ≥48 h after hospital admission or associated with documented in-hospital exposure, and not present or incubating at the time of admission.

SARS-CoV-2 infection was classified as a hospital-acquired (nosocomial) infection when a positive reverse transcription polymerase chain reaction (RT-PCR) test was obtained ≥48 h after admission or when epidemiological evidence supported in-hospital transmission. This classification was adopted to reflect the specific context of the COVID-19 pandemic, while acknowledging the distinct transmission dynamics of viral infections compared to classical bacterial HAIs. Although SARS-CoV-2 infection differs fundamentally from classical bacterial HAIs in terms of transmission routes, risk factors, and pathophysiology, it was included in this analysis to reflect the spectrum of HAIs encountered during the COVID-19 pandemic and to allow comparison of clinical outcomes across major infection categories within the same hospital setting. Community-acquired SARS-CoV-2 infections identified at admission were not included in the analysis.

CDI diagnosis was established based on clinical presentation (diarrhea) and microbiological confirmation, including glutamate dehydrogenase (GDH) antigen detection and toxin A/B assays. The majority of cases were diagnosed using laboratory-based methods, while a small number of cases (*n* = 3) were confirmed by colonoscopy in the appropriate clinical context.

Other bacterial infections were defined as microbiologically confirmed infections classified according to pathogen type and infection site. Due to the limited sample size, this subgroup was included for descriptive purposes only and was excluded from comparative statistical analyses.

Antimicrobial susceptibility data were inherently not applicable to SARS-CoV-2 infection, as viral pathogens do not undergo antibiotic susceptibility testing. For bacterial infections, antimicrobial susceptibility testing results (sensitive/resistant) were available and were used for descriptive microbiological characterization where applicable.

The primary outcome was in-hospital mortality, while secondary outcomes included LOS and time to infection detection.

### 2.6. Microbiological Analysis

Microbiological identification and antimicrobial susceptibility testing were performed using routine laboratory procedures and interpreted according to current European Committee on Antimicrobial Susceptibility Testing (EUCAST) guidelines.

For bacterial infections, antimicrobial susceptibility results were recorded as sensitive or resistant and were used for descriptive characterization of resistance patterns. Due to the limited number of cases and heterogeneity across infection types, antimicrobial resistance data were not included in comparative statistical analyses.

For SARS-CoV-2 infection, diagnosis was based exclusively on RT-PCR testing of nasopharyngeal samples. No antimicrobial susceptibility testing or additional microbiological characterization was applicable, reflecting the viral etiology of the infection.

### 2.7. Infection Control and Surveillance Procedures

During the study period, infection surveillance was performed according to institutional infection prevention and control protocols. Patients with suspected or confirmed SARS-CoV-2 infection were managed using isolation procedures, use of personal protective equipment by healthcare workers, and dedicated patient-flow pathways during the pandemic period. SARS-CoV-2 testing was performed according to hospital policy, which evolved during the pandemic and included testing based on symptoms, epidemiological exposure, and pre-admission or in-hospital screening requirements when applicable. CDI cases were managed according to contact precautions and institutional protocols for diarrheal illness, including laboratory confirmation and infection control notification.

During the study period, infection prevention and control measures were implemented according to institutional protocols and national recommendations in place during the COVID-19 pandemic. These included patient triage (including pre-admission screening), mandatory use of personal protective equipment (PPE) by healthcare workers, mask use for patients, temperature screening at admission, structured patient-flow pathways, and enhanced environmental cleaning and disinfection procedures.

These measures were adapted over time in response to evolving national guidance. However, due to the retrospective design, detailed data on adherence, frequency of implementation, and individual-level exposure to these measures were not consistently available and were therefore not included as analytical variables.

### 2.8. Surgical Admissions and Population at Risk

To contextualize the burden of healthcare-associated infections (HAIs), data on the total number of hospital admissions in each surgical department were collected for the entire study period. These data were used to estimate the relative distribution of HAI cases across departments and to provide a denominator for interpreting infection frequency in relation to patient volume.

Given the retrospective design and variability in case-mix across departments, these data were used for descriptive purposes only and were not incorporated into formal incidence rate calculations.

### 2.9. Statistical Analysis

Statistical analyses were performed using R software 4.5 version (R Foundation for Statistical Computing, Vienna, Austria). Continuous variables were summarized as mean ± standard deviation or median with interquartile range (IQR), depending on distribution, while categorical variables were presented as counts and percentages.

Given the small size of the “other bacterial infections” subgroup (*n* = 12), comparative statistical analyses across all three infection categories were not performed. This subgroup was included for descriptive microbiological characterization only.

Comparative analyses were restricted to the two main groups (SARS-CoV-2 infection and CDI), using the Wilcoxon rank-sum test for continuous variables and chi-square or Fisher’s exact tests for categorical variables, as appropriate.

Univariate logistic regression analysis was performed to identify factors associated with in-hospital mortality. To avoid model overfitting given the limited number of outcome events, the multivariable logistic regression model was restricted to a small number of clinically relevant predictors, including age, overall comorbidity burden, and infection type (SARS-CoV-2 vs. CDI).

Variables such as LOS and time to infection detection were not included in the multivariable model due to their potential role as post-exposure variables and risk of reverse causation.

Time-to-event analysis was performed using Kaplan–Meier survival curves, where time was defined as duration of hospitalization and the event was in-hospital death, with discharge alive treated as censoring. Differences between survival curves were assessed using the log-rank test.

A two-sided *p*-value < 0.05 was considered statistically significant.

## 3. Results

### 3.1. Subsection

#### Cohort Overview

A total of 170 patients with HAIs were included in the study. The mean age of the cohort was 68.78 ± 14.35 years. The median LOS was 21 days (IQR: 17 days), while the median time to infection detection was 14 days. Overall, in-hospital mortality was 15.9% (27 patients) (Table 1). Of these, 85 patients had SARS-CoV-2 infection, 73 had CDI, and 12 had other microbiologically confirmed bacterial infections, which were included for descriptive analysis only.

**Figure 1:**
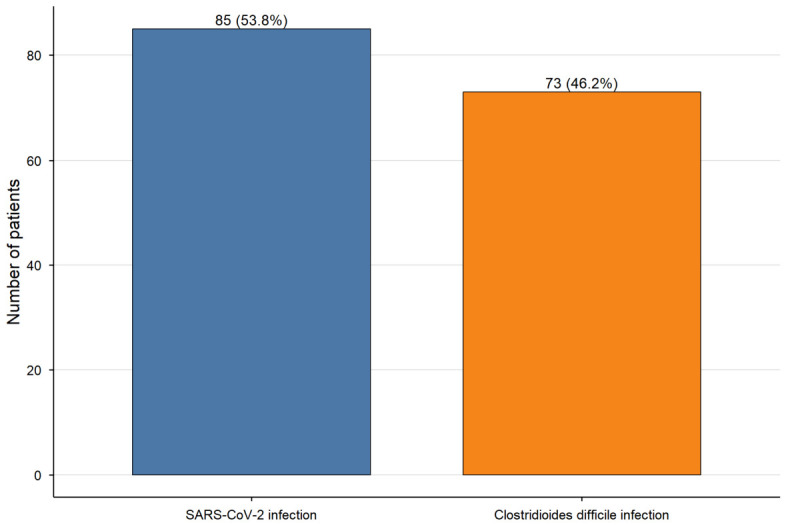
Distribution of infection categories included in the primary comparative analysis. The bar chart shows the number and proportion of patients with SARS-CoV-2 infection (85 cases, 53.8%) and CDI (73 cases, 46.2%). Other bacterial infections (*n* = 12) were excluded from comparative analyses due to the small sample size and are presented descriptively only.

Regarding infection categories, SARS-CoV-2 infection was the most frequent, accounting for 85 cases (53.8%), followed by CDI (73 cases, 46.2%) (Figure 1). Other bacterial infections (*n* = 12) were identified but were excluded from comparative analyses due to the small sample size and are presented descriptively only.

### 3.2. Baseline Characteristics of the Study Groups (SARS-CoV-2 vs. CDI)

Baseline characteristics of patients with SARS-CoV-2 and CDI are presented in Table 2. No significant difference in age was observed between the two groups. Patients with CDI had a significantly higher comorbidity burden and longer hospital stay compared to those with SARS-CoV-2 infection. Time to infection detection did not differ significantly between groups.

Comorbidity burden was higher among patients with CDI, who had a median of 2 comorbidities, compared to a median of 1 comorbidity in patients with SARS-CoV-2 infection. Sex distribution was comparable between the two groups, with a slight predominance of male patients in both SARS-CoV-2 and CDI groups (Table 3).

In-hospital mortality was comparable between patients with SARS-CoV-2 infection (17.6%) and those with CDI (16.4%) (Table 4).

### 3.3. Distribution Across Surgical Departments

The distribution of infections across surgical departments is presented in Table 5. SARS-CoV-2 infection was more frequently observed in orthopedic and general surgery wards, whereas CDI cases were relatively more common in vascular surgery and urology departments. However, these findings should be interpreted with caution due to the small number of cases in several departments. No formal statistical comparisons were performed for this analysis.

**Figure 2:**
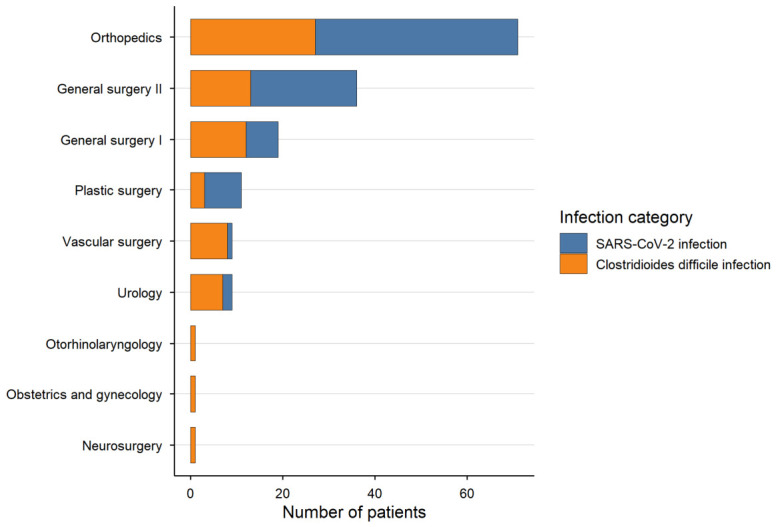
Distribution of SARS-CoV-2 and CDI across surgical departments. The bar chart shows the number of cases (n) for each infection category within each department. SARS-CoV-2 infection was more frequently observed in orthopedic and general surgery wards, whereas CDI cases were relatively more common in vascular surgery and urology departments. These patterns should be interpreted with caution due to the small number of cases in several departments, and no formal statistical comparisons were performed.

Certain departments, including neurosurgery, otorhinolaryngology, and obstetrics and gynecology, reported exclusively CDI cases; however, the number of observations in these departments was very limited (Figure 2).

### 3.4. Comparison Across Infection Categories

#### 3.4.1. Continuous Variables

Significant differences between SARS-CoV-2 CDI groups were observed for LOS, time from admission to infection detection, and comorbidity burden (Table 6). Patients with CDI had a longer median hospital stay compared to those with SARS-CoV-2 infection (23.0 vs. 17.0 days, *p* = 0.015). Time from admission to infection detection was slightly shorter in the CDI group (13.0 vs. 14.0 days, *p* = 0.016). Additionally, patients with CDI presented a higher comorbidity burden (median 2.0 vs. 1.0, *p* < 0.001) (Table 6).

No statistically significant difference in age was observed between the two groups (median 69.0 vs. 71.0 years, *p* = 0.422), although patients in both groups were predominantly elderly.

**Figure 3:**
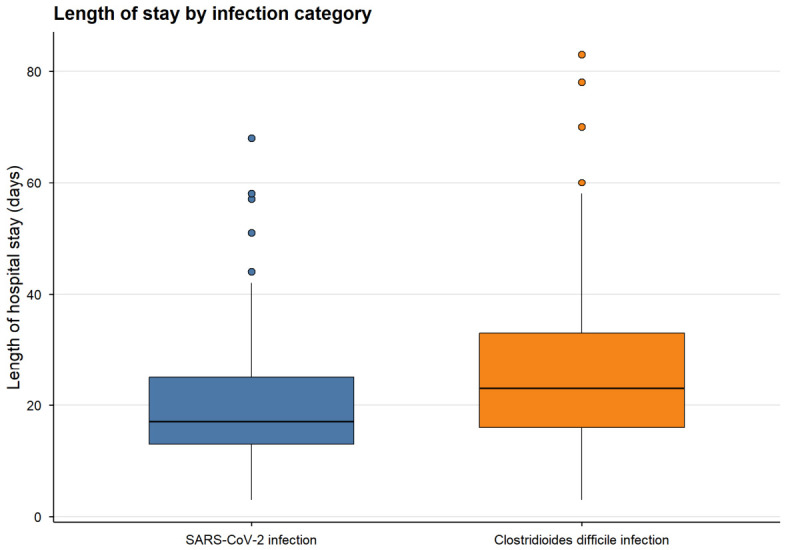
Length of hospital stay by infection group. Boxplots illustrate the distribution of hospital length of stay in patients with SARS-CoV-2 infection and CDI. The central line represents the median, boxes indicate the interquartile range (IQR), and whiskers represent the range excluding outliers.

LOS differed significantly between groups, with longer stays observed in patients with CDI compared to those with SARS-CoV-2 infection (median 23.0 vs. 17.0 days, *p* = 0.015) (Figure 3).

**Figure 4:**
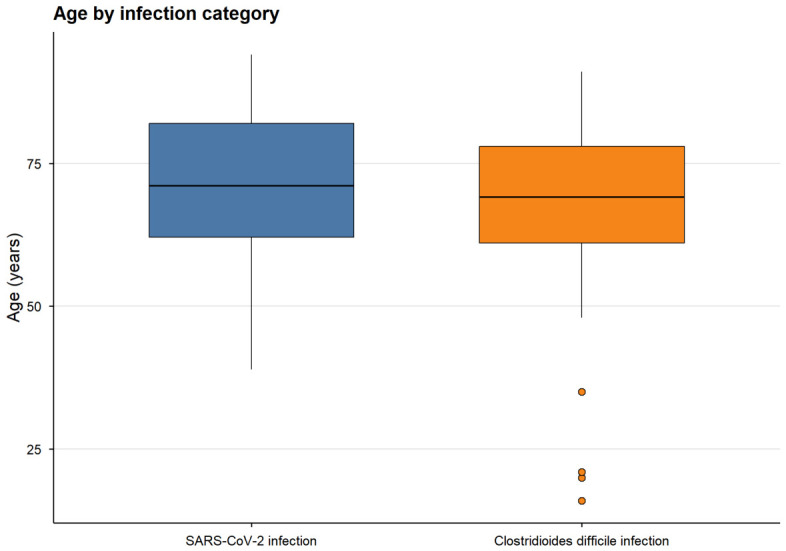
Age distribution by infection group. Boxplots illustrate the distribution of patient age in SARS-CoV-2 and CDI groups. No statistically significant differences were observed between groups. The central line represents the median, boxes indicate the interquartile range (IQR), and whiskers represent the range excluding outliers.

Time from admission to infection detection also differed between groups, with slightly shorter times observed in the CDI group compared to SARS-CoV-2 infection (median 13.0 vs. 14.0 days, *p* = 0.016). LOS was significantly longer in patients with other bacterial infections (median 42.5 days) compared to those with SARS-CoV-2 infection (median 17.0 days) and CDI (median 23.0 days) (*p* < 0.001) (Figure 4).

Comorbidity burden was significantly higher in patients with CDI compared to those with SARS-CoV-2 infection (median 2.0 vs. 1.0, *p* < 0.001).

#### 3.4.2. Categorical Variables

No statistically significant differences were observed between SARS-CoV-2 and CDI groups in terms of sex distribution (*p* = 0.842) or living environment (*p* = 1.000). In contrast, the presence of comorbidities differed significantly between groups (*p* = 0.002), with a higher proportion of patients with CDI presenting at least one comorbidity. In-hospital mortality did not differ significantly between groups (*p* = 1.000) (Table 7).

### 3.5. Microbiological Findings

Among patients with SARS-CoV-2 infection (*n* = 85), diagnosis was established based on RT-PCR testing of nasopharyngeal samples, in accordance with institutional protocols during the study period. As SARS-CoV-2 is a viral pathogen, no microbiological characterization in terms of antimicrobial susceptibility testing was applicable. Clinical presentation was heterogeneous, ranging from asymptomatic infection to cases with radiologically confirmed pneumonia, reflecting the variability of COVID-19 manifestations during the pandemic period.

Among patients with CDI (*n* = 73), diagnosis was established predominantly using laboratory-based methods (95.9%), while colonoscopy was performed in a minority of cases (4.1%).

Regarding diagnostic markers, GDH positivity was identified in 64 cases, toxin A in 66 cases, and toxin B in 15 cases. The higher frequency of toxin A detection compared to toxin B may reflect either strain-related variability or differences in assay sensitivity.

Microbiological and antimicrobial susceptibility data were available primarily for the bacterial infection subgroup. Due to the limited number of cases and heterogeneity of pathogens, comparative analysis across infection categories was not feasible. Descriptive analysis indicated high susceptibility to carbapenems (100% for imipenem, meropenem, and ertapenem) and aminoglycosides (85.7% for amikacin and gentamicin). In contrast, reduced susceptibility was observed for beta-lactams, particularly amoxicillin–clavulanate (0%) and selected cephalosporins (cefuroxime 20%, ceftazidime 40%, cefotaxime 33.3%). Fluoroquinolones showed variable susceptibility, with higher activity for levofloxacin (77.8%) and more balanced profiles for ciprofloxacin and moxifloxacin (~50%).

Overall, these findings indicate a strong reliance on routine laboratory diagnostics, with limited use of endoscopic confirmation. The predominance of toxin-positive cases supports the microbiological confirmation of CDI in this cohort and suggests a consistent diagnostic approach across patients.

### 3.6. Antibiotic Exposure and Associated Infections

Antibiotic exposure prior to diagnosis was observed in 27.4% of patients with CDI. No antibiotic use at admission was recorded in this group. Additionally, 17.8% of CDI cases were associated with other infections. SARS-CoV-2 coinfection was identified in 6.8% of CDI patients. No antibiotic exposure within the previous 60 days or at admission was recorded.

Given these differences in prior antibiotic exposure, subsequent analyses were performed to assess their association with clinical outcomes.

### 3.7. Clinical Outcomes

#### 3.7.1. In-Hospital Mortality and Crude Outcomes

Overall, in-hospital mortality was 15.9% (27/170 patients). Mortality rates were comparable between patients with SARS-CoV-2 infection (17.6%) and CDI (16.4%), while no deaths were recorded among patients with other bacterial infections.

Patients with other bacterial infections had longer hospital stays (median 42.5 days) compared to those with SARS-CoV-2 infection (median 17.0 days) and CDI (median 23.0 days). Similarly, time to infection detection was longest in the other bacterial infections group (median 20.0 days), followed by SARS-CoV-2 (14.0 days) and CDI (13.0 days).

#### 3.7.2. Comparison Between Survivors and Non-Survivors

No statistically significant differences were observed between survivors and non-survivors in terms of age (*p* = 0.089), LOS (*p* = 0.853), or time to infection detection (*p* = 0.709). However, the number of comorbidities was significantly higher among non-survivors (*p* = 0.013), suggesting a greater burden of underlying disease in patients with fatal outcomes.

#### 3.7.3. Predictors of In-Hospital Mortality

In univariate logistic regression analysis, acute respiratory failure (OR 12.30, 95% CI 4.42–36.32, *p* < 0.001), chronic kidney disease (OR 4.735, 95% CI 1.269–17.083, *p* = 0.016), prior hospitalization within the previous 60 days (OR 3.081, 95% CI 1.214–7.611, *p* = 0.015), and a higher number of comorbidities (OR 1.437, 95% CI 1.077–1.927, *p* = 0.014) were significantly associated with in-hospital mortality (Table 8). Age showed a borderline association (*p* = 0.061), while diabetes mellitus, antibiotic exposure, and time to infection detection were not significantly associated with mortality.

In multivariable logistic regression analysis, none of the evaluated variables remained independently associated with in-hospital mortality (Table 9).

#### 3.7.4. Survival Analysis

**Figure 5:**
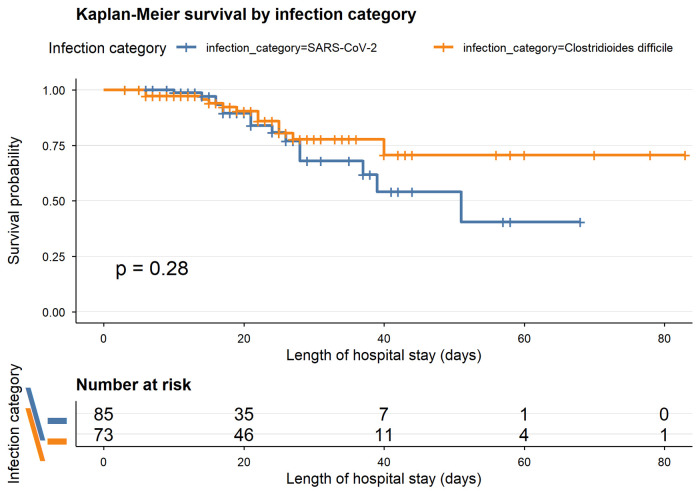
Kaplan–Meier survival curves for patients with SARS-CoV-2 infection and CDI. The log-rank test did not show a statistically significant difference between groups (*p* = 0.28). The number of patients at risk over time is displayed below the plot.

Kaplan–Meier survival analysis did not show a statistically significant difference in survival between patients with SARS-CoV-2 infection and those with CDI (log-rank *p* = 0.28). However, patients with SARS-CoV-2 infection showed a more pronounced decline in survival probability over time compared to those with CDI (Figure 5).

In Cox proportional hazards analysis, chronic kidney disease was independently associated with increased mortality risk (HR 3.752, 95% CI 1.305–10.784, *p* = 0.014). Other variables, including infection category and age, were not significantly associated with survival (Table 10).

## 4. Discussion

In this study, we evaluated clinical outcomes and risk factors associated with HAIs, focusing on SARS-CoV-2 infection and CDI. The main findings were: (i) comparable in-hospital mortality between the two groups, (ii) a significantly higher comorbidity burden in CDI patients, and (iii) clinically relevant predictors identified in univariate analysis that were not retained after adjustment.

SARS-CoV-2 infection represented the largest subgroup, followed by CDI, reflecting the epidemiological impact of the COVID-19 pandemic. This distribution should also be interpreted in the context of reduced elective surgical activity and altered patient flow, which likely decreased the incidence of typical surgical HAIs, such as surgical site infections, while increasing the relative contribution of SARS-CoV-2–related cases.

Secondary infections in COVID-19 patients are associated with prolonged hospitalization and worse outcomes, particularly in critically ill populations [9,20]. Despite distinct etiologies, in-hospital mortality was comparable between SARS-CoV-2 infection (17.6%) and CDI (16.4%), consistent with previous reports highlighting substantial mortality in both conditions, especially among elderly and comorbid patients [3,21].

Differences in hospital LOS likely reflect distinct clinical trajectories. COVID-19 patients often have shorter or more standardized hospitalizations, particularly outside ICU settings [22], whereas CDI is associated with prolonged stays due to complications and recurrence risk [23]. Similarly, HAIs and MDR infections increase LOS due to more complex management [24,25]. In our cohort, this difference may also reflect the timing of infection onset: SARS-CoV-2 was frequently identified early during admission, whereas CDI typically develops during hospitalization. In addition, pandemic-related changes in hospital organization, including prioritization of bed availability and standardized care pathways, may have contributed to shorter and more homogeneous stays in COVID-19 patients.

CDI patients presented a significantly higher comorbidity burden, consistent with its pathogenesis involving immune dysfunction, microbiota disruption, and healthcare exposure [3,5]. Comorbidity burden was also associated with mortality in univariate analysis, supporting previous evidence that cumulative clinical vulnerability is a key determinant of adverse outcomes [26]. In contrast, demographic factors such as sex and living environment were not associated with infection category, suggesting that clinical factors outweigh baseline demographic variability [27].

Survival analysis provided additional nuance. Kaplan–Meier curves did not show statistically significant differences (log-rank *p* = 0.28), although a steeper decline in survival probability was observed in the SARS-CoV-2 group. In univariate analysis, acute respiratory failure, chronic kidney disease, recent hospitalization, and comorbidity burden were associated with mortality, with acute respiratory failure showing the strongest effect.

However, none of these variables remained significant in the multivariable model. This discrepancy likely reflects limited statistical power due to a small number of outcome events, potential overfitting, and collinearity between clinically related variables such as comorbidity burden and disease severity. In addition, variables such as LOS and time to infection detection may act as intermediate rather than baseline predictors, complicating their interpretation in adjusted models. Similar discrepancies have been reported in heterogeneous clinical cohorts [28]. Therefore, the absence of independent predictors should be interpreted with caution and does not negate their clinical relevance, but rather highlights the need for larger, adequately powered studies.

Our findings should also be interpreted in the broader context of AMR. Increasing evidence highlights the growing burden of multidrug-resistant organisms and their impact on outcomes [16]. In our cohort, prior antibiotic exposure was observed in 27.4% of CDI patients, supporting the role of antibiotic-driven dysbiosis and providing a rationale for targeted antimicrobial stewardship.

Microbiological and antimicrobial susceptibility data were available primarily for the bacterial infection subgroup. Although resistance profiles were recorded at the isolate level, the limited number of cases and heterogeneity of pathogens precluded a robust analysis across infection categories. Nevertheless, exploratory analysis suggested class-specific resistance patterns, with preserved activity of carbapenems and aminoglycosides, and reduced susceptibility to beta-lactams, particularly amoxicillin-clavulanate and selected cephalosporins, while fluoroquinolones showed variable activity. These observations, although limited, support the importance of pathogen-guided antimicrobial strategies over uniform empirical approaches. Bloodstream infections caused by resistant pathogens, including *P. aeruginosa*, remain associated with high mortality, particularly in patients with severe comorbidities [29].

Although a detailed AMR analysis was beyond the scope of this study, the observed patterns are consistent with broader evidence linking antimicrobial resistance to increased disease severity, recurrence, and prolonged hospitalization [12,20,30,31].

From a clinical perspective, the comparable mortality between SARS-CoV-2 infection and CDI highlights CDI as a persistent contributor to in-hospital mortality. The higher comorbidity burden among CDI patients supports the need for targeted risk stratification and closer monitoring, while the association between acute respiratory failure and mortality emphasizes the importance of early recognition and management.

From an infection control perspective, our findings suggest that HAI prevention strategies should integrate both pathogen-specific and patient-related risk factors. The association between prior hospitalization and adverse outcomes highlights the importance of continuity in infection prevention measures, including antimicrobial stewardship and post-discharge monitoring. In addition, targeted interventions such as antibiotic stewardship programs, early identification of high-risk patients (particularly those with chronic kidney disease), and enhanced CDI surveillance may improve outcomes.

The observed infection distribution should also be interpreted in the context of infection prevention measures implemented during the COVID-19 pandemic, including patient triage, PPE use, structured patient flow, and enhanced hygiene protocols. Although not quantitatively assessed, these measures likely contributed to the reduced occurrence of typical surgical HAIs and the altered infection profile observed.

This study has several limitations. The retrospective single-center design may limit generalizability and introduce selection bias. The small size of the bacterial infection subgroup restricts interpretability and reflects the pandemic context, characterized by reduced elective surgical activity and altered patient flow. To avoid underpowered comparisons, this subgroup was excluded from primary analyses and used descriptively. Consequently, the absence of mortality or lower morbidity in this group should be interpreted cautiously. The limited number of outcome events may also have reduced statistical power, particularly in multivariable models. In addition, incomplete microbiological and AMR data across all groups limited pathogen-specific interpretation.

Despite these limitations, the study provides a comprehensive evaluation of clinical outcomes across infection categories within a unified cohort. The integration of descriptive, regression, and survival analyses enables a multidimensional assessment of mortality risk, enhancing the clinical relevance of the findings.

## 5. Conclusions

In conclusion, in-hospital mortality was comparable between patients with SARS-CoV-2 infection and CDI, confirming the substantial clinical burden of CDI in hospitalized populations. CDI patients presented a significantly higher comorbidity burden, which also emerged as a key determinant of adverse outcomes.

In univariate analysis, acute respiratory failure, chronic kidney disease, prior hospitalization, and comorbidity burden were associated with mortality, although these associations did not persist in multivariable models, likely due to collinearity and limited statistical power. In survival analysis, chronic kidney disease remained independently associated with increased mortality risk.

These findings also emphasize the need for targeted infection control strategies that integrate comorbidity burden and clinical vulnerability, rather than relying exclusively on pathogen-based approaches.

These findings highlight the need for targeted infection prevention strategies, particularly in patients with increased comorbidity burden, as well as careful antibiotic use given the observed association between prior antibiotic exposure and CDI.

Further multicenter studies with larger cohorts and detailed microbiological data are needed to validate these findings and refine prognostic models in patients with HAIs.

## Figures and Tables

**Table 1:** Overall characteristics of the study cohort.

Total Patients	Mean Age (Years)	SD	Median LOS (Days)	IQR	Mortality, n	Mortality, %
170	68.78	14.35	21	17	27	15.9

Data are presented as mean ± standard deviation (SD) or median (interquartile range, IQR), as appropriate. LOS = length of stay.

**Table 2:** Baseline characteristics of patients with SARS-CoV-2 and CDI included in the comparative analysis.

Infection Category	*n*	Age (Years)	Length of Stay (Days)	Time to Infection Detection (Days)	Comorbidity Burden
SARS-CoV-2	85	71.00 (62.0–82.0)	17.00 (13.0–25.0)	14.00 (11.0–19.0)	1.00 (0.0–1.0)
*C. difficile*	73	69.00 (61.0–78.0)	23.00 (16.0–33.0)	13.00 (8.0–17.0)	2.00 (1.0–3.0)

Continuous variables are presented as median (interquartile range, IQR). *p*-values were calculated using the Mann–Whitney U test.

**Table 3:** Sex distribution by infection group (SARS-CoV-2 vs. CDI).

Sex	SARS-CoV-2 Infection	*C. difficile* Infection
F	36 (42.4%)	33 (45.2%)
M	49 (57.6%)	40 (54.8%)

**Table 4:** In-hospital mortality by infection group (SARS-CoV-2 vs. CDI).

Outcome	SARS-CoV-2 Infection	*C. difficile* Infection
Survived	70 (82.4%)	61 (83.6%)
Died	15 (17.6%)	12 (16.4%)

Data are presented as number (percentage). No deaths were recorded in the “Other bacterial infections” group.

**Table 5:** Distribution of SARS-CoV-2 and CDI across surgical departments.

Department	SARS-CoV-2 (n, %)	CDI (n, %)	Total (n)
Orthopedics	44 (62.0%)	27 (38.0%)	71
General Surgery II	23 (63.9%)	13 (36.1%)	36
General Surgery I	7 (36.8%)	12 (63.2%)	19
Plastic Surgery	8 (72.7%)	3 (27.3%)	11
Urology	2 (22.2%)	7 (77.8%)	9
Vascular Surgery	1 (11.1%)	8 (88.9%)	9
Neurosurgery	0	1	1
Obstetrics and Gynecology	0	1	1
Otorhinolaryngology	0	1	1

Data are presented as number of cases (*n*) and percentage within each department. CDI, *Clostridioides difficile* infection.

**Table 6:** Comparison of continuous variables between SARS-CoV-2 and CDI groups.

Variable	SARS-CoV-2 Infection	CDI	*p*-Value
Age	70.07 ± 13.47; median 71.00 (IQR 62.00 to 82.00)	67.55 ± 15.20; median 69.00 (IQR 61.00 to 78.00)	0.422
Length of hospital stay	20.92 ± 12.42; median 17.00 (IQR 13.00 to 25.00)	26.05 ± 16.06; median 23.00 (IQR 16.00 to 33.00)	0.015
Time to infection detection	16.59 ± 9.48; median 14.00 (IQR 11.00 to 19.00)	13.97 ± 11.44; median 13.00 (IQR 8.00 to 17.00)	0.016
Number of comorbidities	1.01 ± 1.25; median 1.00 (IQR 0.00 to 1.00)	1.77 ± 1.36; median 2.00 (IQR 1.00 to 3.00)	<0.001

Interquartile range, IQR; *p*-values were calculated using the Mann–Whitney U test.

**Table 7:** **Comparison of categorical variables between SARS-CoV-2 and CDI groups**.

Variable	Level	SARS-CoV-2 Infection (*n* = 85)	CDI (*n* = 73)	*p*-Value
Sex	Female	36 (42.4%)	33 (45.2%)	0.842
	Male	49 (57.6%)	40 (54.8%)	
Living environment	Urban	46 (54.1%)	40 (54.8%)	0.01
	Rural	39 (45.9%)	33 (45.2%)	
Presence of comorbidities	No	37 (43.5%)	14 (19.2%)	0.02
	Yes	48 (56.5%)	59 (80.8%)	
In-hospital mortality	Survived	70 (82.4%)	61 (83.6%)	1.00
	Died	15 (17.6%)	12 (16.4%)	

Data are presented as number (percentage). *p*-values were calculated using the chi-square or Fisher’s exact test, as appropriate.

**Table 8:** Univariate logistic regression analysis for in-hospital mortality.

Variable	OR	95% CI	*p*-Value
Acute respiratory failure	12.30	4.42–36.32	<0.001
Chronic kidney disease	4.735	1.269–17.083	0.016
Hospitalization (past 60 days)	3.081	1.214–7.611	0.015
Number of comorbidities	1.437	1.077–1.927	0.014
Age	1.033	1.000–1.072	0.061
Diabetes mellitus	1.977	0.740–4.958	0.156
Antibiotic exposure (past 60 days)	2.190	0.575–7.930	0.232
Time to infection detection (days)	1.007	0.966–1.044	0.735

OR: odds ratio; CI: confidence interval.

**Table 9:** Multivariable logistic regression analysis of factors associated with in-hospital mortality.

Variable	OR	95% CI	*p*-Value
(Intercept)	0.003	0.00–0.219	0.027
Age	1.049	0.988–1.136	0.173
Diabetes mellitus	2.638	0.557–12.484	0.211
Chronic kidney disease	4.390	0.806–25.037	0.084
Heart failure	0.305	0.015–2.042	0.296
Chronic obstructive pulmonary disease	0.726	0.029–7.554	0.806
Antibiotic exposure (past 60 days)	2.725	0.512–14.861	0.144

OR: odds ratio; CI: confidence interval. Variables included in the multivariable model were selected based on clinical relevance and univariate analysis.

**Table 10:** Cox proportional hazards analysis.

Variable	HR	CI	*p*-Value
CDI	0.517	0.217 to 1.231	0.136
Age	1.032	0.995 to 1.069	0.088
Diabetes mellitus	1.490	0.6 to 3.701	0.390
Chronic kidney disease	3.752	1.305 to 10.784	0.014
Heart failure	1.117	0.367 to 3.401	0.845
Chronic obstructive pulmonary disease	0.916	0.187 to 4.481	0.914

HR: hazard ratio; CI: confidence interval; other bacterial infections were excluded from the Cox model due to insufficient events.

## Data Availability

The original contributions presented in this study are included in the article. Further inquiries can be directed to the corresponding author.

## Abbreviations

The following abbreviations are used in this manuscript:

HAIs	Healthcare-associated infections
AMR	Antimicrobial resistance
CDI	*Clostridioides difficile* infection
COVID-19	Coronavirus disease 2019
SARS-CoV-2	Severe acute respiratory syndrome coronavirus 2
GDH	Glutamate dehydrogenase
EUCAST	European Committee on Antimicrobial Susceptibility Testing
IQR	Interquartile range
HR	Hazard ratio
SD	Standard deviation
OR	Odds ratio
CI	Confidence interval

## References

[1] Ricchizzi E., Sasdelli E., Leucci A.C., Fabbri E., Caselli L., Latour K., Panis L.I., De Baets E., Abeele A.-M.V.D., D’AMbrosio A. (2025). Incidence of health-care-associated infections in long-term care facilities in nine European countries: A 12-month, prospective, longitudinal cohort study. Lancet Infect. Dis..

[2] Sands K.E., Blanchard E.J., Fraker S., Korwek K., Cuffe M. (2023). Healthcare-associated infections among hospitalized patients with COVID-19, March 2020–March 2022. JAMA Netw. Open.

[3] Stămăteanu L.O., Miftode I.L., Pleşca C.E., Hurmuzache M.E., Manciuc D.C., Leca D., Miftode E.G. (2025). Healthcare-associated *Clostridioides difficile* infection: A hospital-based retrospective study in North Eastern Romania. Microorganisms.

[4] Gagliano M.C., D’Agati G., Medaglia A.A., Pipitò L., Catania B., Conti C., Tuttolomondo A., Cefalù A.B., Cammà C., Scichilone N. (2025). Burden of *Clostridioides difficile* infection and risk factors for recurrences in an Italian tertiary care university hospital: A prospective observational study. Antibiotics.

[5] Borji S., Rostamian M., Kadivarian S., Kooti S., Dashtbin S., Hosseinabadi S., Abiri R., Alvandi A. (2022). Prevalence of *Clostridioides difficile* contamination in the healthcare environment and instruments: A systematic review and meta-analysis. Germs.

[6] Rabiee M.H., Abdolmohammadi Khiav L., Fallah Mehrabadi M.H. (2026). Prevalence of *Clostridioides difficile* contamination in healthcare and non-healthcare environments: A global systematic review and meta-analysis. Int. Health.

[7] Jabłońska R., Sokal P., Zając M., Królikowska A., Filipska-Blejder K., Wrońska I., Ślusarz R. (2025). Epidemiology and microbiology of healthcare-associated infections in neurosurgery department: A cross-sectional study. Biol. Res. Nurs..

[8] Odoom A., Donkor E.S. (2025). Prevalence of healthcare-acquired infections among adults in intensive care units: A systematic review and meta-analysis. Health Sci. Rep..

[9] Binkhamis K., Alhaider A.S., Sayed A.K., Almufleh Y.K., Alarify G.A., Alawlah N.Y. (2023). Prevalence of secondary infections and association with mortality rates of hospitalized COVID-19 patients. Ann. Saudi Med..

[10] Sahrmann J.M., Nickel K.B., Stwalley D., Dubberke E.R., Lyons P.G., Michelson A.P., McMullen K.M., Gandra S., Olsen M.A., Kwon J.H. (2023). Healthcare-associated infections (HAIs) during the coronavirus disease 2019 (COVID-19) pandemic: A time-series analysis. Antimicrob. Steward. Healthc. Epidemiol..

[11] Khavandegar A., Siami Z., Rasouli A., Nazemi P., Gull A. (2025). Impact of healthcare-associated infections on in-hospital outcomes during the COVID-19 era: A multicenter comparative study of 20,942 isolated microorganisms from ICU patients. Front. Public Health.

[12] Peconi C., Martini E., Sarti D., Prospero E. (2025). Impact of the COVID-19 pandemic on healthcare-associated infections and multidrug-resistant microorganisms in Italy: A systematic review. J. Infect. Public Health.

[13] Erayman İ., Kandemir B., Bulut R., Akgül M., Uyar M., Kurt E.K. (2026). Healthcare-associated infections before and during the pandemic: Four-year follow-up. BMC Infect. Dis..

[14] Langlete P., Eriksen-Volle H.M., Paulsen T.H., Raastad R., Fagernes M., Bøås H., Himmels J. (2025). Healthcare-associated COVID-19 infections and mortality. J. Hosp. Infect..

[15] Voinea C., Mocanu E., Dantes E., Jurja S., Neculai A.-M., Craciun A., Rugina S. (2025). Burden of healthcare-associated infections on mortality among COVID-19 hospitalized patients. J. Clin. Med..

[16] Borcan A.M., Radu G., Simoiu M., Costea E.L., Rafila A. (2024). A five-year analysis of antibiotic resistance trends among bacteria identified in positive urine samples in a tertiary care hospital from Bucharest, Romania. Antibiotics.

[17] Budianu M.-A., Ciurea C.N., Moraru L., Voidăzan S. (2025). Burden of healthcare-associated infections and antimicrobial resistance in a Romanian cardiovascular and transplant center: Factors associated with mortality. Antibiotics.

[18] Ciccacci F., De Santo C., Mosconi C., Orlando S., Carestia M., Guarente L., Liotta G., Palombi L., Gialloreti L.E. (2024). Not only COVID-19: A systematic review of anti-COVID-19 measures and their effect on healthcare-associated infections. J. Hosp. Infect..

[19] (2025). Research on the prevalence and characteristics of HAI in surgical departments within the Pitesti County Emergency Hospital.

[20] Hu G.N., Liu W.L., Chang C.H., Ruan S.Y., Chung K.P., Chien J.Y., Yu C.-J. (2024). Microbial dynamics, risk factors and outcomes of secondary pneumonia in critically ill patients with COVID-19: A multicenter retrospective cohort study. J. Formos. Med. Assoc..

[21] Fabbri A., Tascioglu A.B., Bertini F., Benazzi B., Montesi D. (2026). Healthcare-associated infections impact mortality in patients admitted to the acute care hospital from the emergency department. J. Clin. Med..

[22] Garbin J.R.T., Leite F.M.C., Santos A.P.B.D., Dell’Antonio L.S., Dell’Antonio C.S.D.S., Lopes-Júnior L.C. (2025). Factors associated with prolonged hospitalizations for COVID-19 during the first three waves of the pandemic: Evidence from a Southeastern State of Brazil. PLoS ONE.

[23] Drobnik J., Pobrotyn P., Moricová Š., Madziarska K., Baran M. (2024). Analysis of factors affecting the length of hospitalization of patients with Clostridioides difficile infection: A cross-sectional study. Arch. Public Health.

[24] Chen Y.P., Tasi X.W., Chang K., Cao X.D., Chen J.R., Liao C.S. (2021). Multi-Drug Resistant Organisms Infection Impact on Patients Length of Stay in Respiratory Care Ward. Antibiotics.

[25] Moradi S., Najafpour Z., Cheraghian B., Keliddar I., Mombeyni R. (2024). The Extra Length of Stay, Costs, and Mortality Associated With Healthcare-Associated Infections: A Case-Control Study. Health Sci. Rep..

[26] Feng H., Zhou F., Shen Y., Wang Z., Yuan Y., Jing W., Zheng Z., Peng H., Yu Q. (2025). Prognostic value of the Charlson Comorbidity Index for mortality and machine learning-based prediction in critically ill patients with paralytic ileus: Retrospective cohort study. JMIR Med. Inform..

[27] Yamani L.N., Astutik E., Qurniyawati E., Lusida M.I., Getaneh Y., Kelly M. (2025). Associations between socio-demographics, sexual knowledge and behaviour and sexually transmitted infections among reproductive-age women in Southeast Asia: Demographic Health Survey results. BMC Public Health.

[28] Sandu A.M., Vrancianu C.O., Tantu A.-C., Dumitrache V.M., Diaconescu D., Cristian R.-E., Marcu A., Tantu M.M. (2026). Epidemiology of Healthcare-Associated Infections Caused by Multidrug-Resistant Bacteria and Antimicrobial Resistance Patterns in a Romanian Tertiary Care Hospital. J. Clin. Med..

[29] Derin O., Şahin M., Dumlu R., Başgönül S., Bayrak A.D., Arduç Ş., Bayram S., Mikaliyova N., Kantürk A., Öncül A. (2024). Registry-based retrospective cohort study of mortality among adults admitted to intensive care units in Istanbul with hospital-acquired *Pseudomonas aeruginosa* bloodstream infection between 2014–2021. Antibiotics.

[30] Walker M.K., Yek C., Sarzynski S., Warner S., Harris A.D., Baghdadi J.D., E Goodman K., Powers J.H., Klompas M., Rhee C. (2026). Survival trends in patients with difficult-to-treat, antibiotic-resistant, Gram-negative infections in the era of next-generation antibiotics in the USA: A retrospective cohort study. Lancet Infect. Dis..

[31] Pintea-Simon I.A., Bancu L., Mare A.D., Ciurea C.N., Toma F., Brukner M.C., Văsieșiu A.-M., Man A. (2024). Secondary bacterial infections in critically ill COVID-19 patients admitted in the intensive care unit of a tertiary hospital in Romania. J. Clin. Med..

